# Telehealth Support From Cardiologists to Primary Care Physicians in Heart Failure Treatment: Mixed Methods Feasibility Study of the Brazilian Heart Insufficiency With Telemedicine Trial

**DOI:** 10.2196/64438

**Published:** 2025-04-17

**Authors:** Leonardo Graever, Priscila Cordeiro Mafra, Vinicius Klein Figueira, Vanessa Navega Miler, Júlia dos Santos Lima Sobreiro, Gabriel Pesce de Castro da Silva, Aurora Felice Castro Issa, Leonardo Cançado Monteiro Savassi, Mariana Borges Dias, Marcelo Machado Melo, Viviane Belidio Pinheiro da Fonseca, Isabel Cristina Pacheco da Nóbrega, Maria Kátia Gomes, Laís Pimenta Ribeiro dos Santos, José Roberto Lapa e Silva, Anne Froelich, Helena Dominguez

**Affiliations:** 1 Department of Biomedical Sciences Faculty of Health and Medical Sciences University of Copenhagen Copenhagen Denmark; 2 Departamento de Clínica Médica Faculdade de Medicina Universidade Federal do Rio de Janeiro Rio de Janeiro Brazil; 3 Instituto de Atenção à Saúde São Francisco de Assis Centro de Ciências da Saúde Universidade Federal do Rio de Janeiro Rio de Janeiro Brazil; 4 Instituto Nacional de Cardiologia Rio de Janeiro Brazil; 5 Faculdade de Medicina Universidade Federal Fluminense Niterói Brazil; 6 Faculdade de Medicina Instituto de Educação Médica Rio de Janeiro Brazil; 7 Departamento de Medicina de Família e Comunidade, Saúde Mental e Coletiva Escola de Medicina Universidade Federal de Ouro Preto Ouro Preto Brazil; 8 Ministério da Saúde Brasília Brazil; 9 Escola Nacional de Saúde Pública FIOCRUZ Rio de Janeiro Brazil; 10 Department of Public Health Faculty of Health and Medical Sciences University of Copenhagen Copenhagen Denmark; 11 Department of Cardiology Bispebjerg Hospital Copenhagen Denmark

**Keywords:** heart failure, telemedicine, telehealth, intersectoral collaboration, primary health care, low- and middle-income countries, family practice

## Abstract

**Background:**

Heart failure is a prevalent condition ideally managed through collaboration between health care sectors. Telehealth between cardiologists and primary care physicians is a strategy to improve the quality of care for patients with heart failure. Still, the effectiveness of this approach on patient-relevant outcomes needs to be determined.

**Objective:**

This study aimed to assess the feasibility of telehealth support provided by cardiologists for treating patients with heart failure to primary care physicians from public primary care practices in Rio de Janeiro, Brazil.

**Methods:**

We used mixed methods to assess the feasibility of telehealth support. From 2020 to 2022, we tested 2 telehealth approaches: synchronous videoconferences (phase A) and interaction through an asynchronous web platform (phase B). The primary outcome was feasibility. Exploratory outcomes were telehealth acceptability of patients, primary care physicians, and cardiologists; the patients’ clinical status; and prescription practices. Qualitative methods comprised content analysis of 3 focus groups and 15 individual interviews with patients, primary care physicians, and cardiologists. Quantitative methods included the baseline assessment of 83 patients; a single-arm, before-and-after assessment of clinical status in 58 patients; and an assessment of guideline-directed medical therapy in 28 patients with reduced ejection fraction measured within 1 year of follow-up. We integrated qualitative and quantitative data using a joint display table and used the A Process for Decision-Making After Pilot and Feasibility Trials framework for feasibility assessment.

**Results:**

Telehealth support from cardiologists to primary care physicians was generally well accepted. As barriers, patients expressed concern about reduced direct access to cardiologists, primary care physicians reported work overload and a lack of relative advantage, and cardiologists expressed concern about the sustainability of the intervention. Quantitative analysis revealed an overall poor baseline clinical status of patients with heart failure, with 53% (44/83) decompensated, as expected. Compliance with guideline-directed medical therapy for the treatment of heart failure with reduced ejection fraction after telehealth showed a modest improvement for β-blockers (17/20, 85% to 18/19, 95%) and renin-angiotensin-aldosterone system inhibitors (14/20, 70% to 15/19, 79%) but a drop in the prescription of spironolactone (16/20, 80% to 15/20, 75%). Neprilysin and sodium-glucose cotransporter 2 inhibitors were introduced in 4 and 1 patient, respectively. Missing record data precluded a more precise analysis. The feasibility assessment was positive, favoring the asynchronous modality. Potential modifications include more effective patient and professional recruitment strategies and educational activities to raise awareness of collaborative support in primary care.

**Conclusions:**

Telehealth was feasible to implement. Considering the stakeholders’ views and insights on the process is paramount to attaining engagement. Missing data must be anticipated for future research in this setting. Considering the recommended adaptations, the intervention can be studied in a cluster-randomized trial.

## Introduction

### Background

Collaboration among health care professionals is essential for delivering the best possible care for the population [1]. Telehealth, defined in this paper as the interaction between health care professionals using remote communication tools to collaborate on patient care [2,3], may increase the efficiency of health care systems, reduce costs, and improve patients’ quality of life while lowering the need for in-person appointments with specialists and referrals [4,5]. Specifically, chronic disease management involving multidisciplinary collaboration is known to improve the quality of care [6,7].

Heart failure is a chronic condition and the end stage of many cardiovascular diseases, with a significant impact on public health [8-10]. Recent epidemiologic studies on the global burden of disease point to an incidence of up to 20 cases per 1000 persons per year and a prevalence of 1% to 3% of the population, affecting 64 million people worldwide [11-14]. Readmission rates can be as high as 40% in 6 months [15], burdening health systems with an estimated annual cost of US $108 billion worldwide [16]. The 5-year specific mortality rate may reach 75%, and quality of life is jeopardized. Population aging, the increase in survival rates after acute cardiologic events, and better access to health care will increase the prevalence of heart failure by up to 8.5% in 2030 according to prediction models [17].

Notwithstanding the unfavorable epidemiological scenario, heart failure is amenable to pharmacological treatment and behavior change. Most interventions can be delivered in primary care [18,19] and other outpatient settings with positive results [20,21], and new guidelines, including novel pharmacological options, are published and updated frequently [22,23]. Nevertheless, the overall physician adherence to the recommendations is low. The proportion of patients with heart failure with reduced ejection fraction (HFrEF) treated following guideline-directed medical therapy (GDMT) is reported as 27% to 73%, constituting only 14% when reaching target doses is considered [24]. Primary care physicians with a general medicine background commonly need support in assisting these patients, as described in previous studies [25-28]. Therefore, there is plenty of room for improvement, making it a suitable case for collaborative strategies such as telehealth.

Telehealth services have been commonly used as a collaborative care strategy, mainly in North America and, to a lesser extent, in Europe [29], with positive results [30,31]. They are less common in low- and middle-income countries. Brazil has a national telehealth program named *Telessaúde Brasil Redes* [32], which aims to foster the development of telehealth nuclei in Brazilian states and regions. At least 3 large telehealth services have been implemented in the last decades. Unfortunately, reports about telehealth implementation in Brazil have pointed to low adoption rates by primary care physicians [33-37].

Implementation research studies indicate that telehealth implementation, as a complex intervention, is influenced by multiple factors that may facilitate or undermine its adoption and usability [38-40]. Telehealth adoption is below the expected level in many settings due to subjective factors such as resistance to innovation and practical aspects such as infrastructure availability, technical challenges, communication hardships between sectors, and work overload from other tasks [41-43]. Furthermore, solid, high-quality evidence of the benefit of telehealth, especially in assessing patient-relevant outcomes, is lacking [44]. Recently published systematic reviews point to the need for trials with enough statistical power focusing on patient-relevant outcomes such as mortality, hospital admissions, and quality of life [4,29,44,45]. For all the reasons and knowledge gaps described previously, we designed a clinical trial [46] within the Brazilian Heart Insufficiency With Telemedicine (BRAHIT) frame project, an academic collaboration between medical researchers from Denmark and Brazil’s higher education and health institutions [47]. The trial aims to evaluate whether telehealth support from cardiologists to primary care physicians improves the quality of heart failure management and impacts patient-relevant outcomes.

As recommended by most frameworks for studying complex interventions [48,49], we previously tested the implementation of the intervention used in this study, aiming to assess the feasibility of the telehealth process designed as the trial intervention. We tested a synchronous approach, where real-time case discussions are held between specialists and primary care physicians using remote communication tools (eg, videoconference), and an asynchronous approach, where the communication does not require real-time contact between the parties and the remote interaction happens using a non–real-time strategy (eg, SMS text messages).

We aimed to answer the following research question: is it feasible to implement telehealth support from cardiologists to primary care physicians in the clinical practice settings of Rio de Janeiro and evaluate it as an intervention within a cluster-randomized trial? Other pertinent research questions included the following: which factors influence primary care physicians’ adoption of telehealth support? How do other stakeholders, such as patients and teleconsulting cardiologists, perceive the intervention? Does telehealth support alter current clinical practices among primary care physicians?

### Objectives

This study aimed to analyze factors influencing the delivery and acceptability of telehealth support by primary care physicians, cardiologists, and patients (stakeholders), including context factors, facilitators, barriers, opportunities, and threats, and analyze whether telehealth support influences primary care physicians’ treatment practices and the clinical status of patients with heart failure.

## Methods

### Study Design

This was a prospective study using mixed methods and a concurrent design. The qualitative approach included thematic analysis of data from focus groups and individual interviews with the participants using predefined, semistructured scripts. The analysis followed an inductive, constructivist approach. We sought data about the context and the telehealth execution, drawing connections between our preconceived hypotheses and assumptions (theories) and the collected data guided by the content analysis methodology by Bardin [50]. We chose this design to collect and analyze descriptive and subjective in loco information that could help us answer our research questions. The quantitative assessment involved a descriptive analysis of the patients’ clinical changes, including vital signs, symptoms, and prescribed medications in the cases discussed.

For reporting guidance, we used, where applicable, the CONSORT (Consolidated Standards of Reporting Trials) extension for pilot and feasibility trials [51], the Strengthening the Reporting of Observational Studies in Epidemiology statement for observational research [52], the Standards for Reporting Qualitative Research statement [53], the recommendations by Braun and Clarke [54] for reporting qualitative studies, guidelines for reporting mixed methods studies [55], and additional guiding literature [56,57].

### Setting

The BRAHIT project started in 2019 with the principal aim of implementing digital solutions to improve the quality of cardiovascular disease care in Rio de Janeiro, Brazil’s second-largest city with 6.2 million inhabitants. Brazil’s population relies on a universal health system with free access to comprehensive care, and Brazil has invested in primary care through the implementation of the Family Health Strategy over the last 25 years [58]. In this context, Rio de Janeiro has been the setting for significant primary care reforms in the previous 15 years, showing a marked increase in health care structure and workforce [59]. There are currently 238 primary health care practices in the city hosting 1352 teams, each composed of 1 physician, 1 nurse, 1 nurse technician, and 5 to 6 community health workers. Primary care practices also deliver oral health care and have the support of mental health and rehabilitation professionals.

As one of the main cities in the country and former capital, Rio de Janeiro also hosts a thorough specialized service network, including national institutes such as the National Institute of Cardiology (INC), whose team was responsible for the telehealth support to the primary care teams in this study. The choice of telehealth as the studied intervention within the BRAHIT project relied on the strategic role of collaborative interactions between health services to improve health care [6], which aligned with the project’s main strategic goal.

Other BRAHIT project research activities include a systematic review of telehealth and a cluster-randomized trial registered at ClinicalTrials.gov (NCT04466852), which was in the recruitment phase when this paper was submitted.

### Intervention

#### Overview

The intervention assessed in this study was telehealth support requested by a primary care physician to discuss a heart failure case and executed by a cardiologist from the INC. The intervention aimed to support general physicians in dealing with the clinical aspects of heart failure management, including diagnostic, treatment, and referral practices. The feasibility study and interventions were organized in 2 different phases and approaches. Telehealth occurred through scheduled synchronous videoconferences or an asynchronous texting and data exchange platform depending on the study phase, as described in the following sections.

#### Phase A: Synchronous Videoconferences

Phase A started in August 2020, when videoconferences (synchronous approach) between cardiologists and primary care physicians were implemented to discuss cases of patients with heart failure from one of the Rio de Janeiro municipality’s primary care practices. The practice comprised 15 primary care teams. As one of the hosts of the family medicine residency program in Rio de Janeiro, it also has 2 family medicine residents per team (year 1 and year 2) in addition to the original team composition described previously. This practice provides primary care for >45,000 people in a socioeconomically deprived area.

The research team presented the BRAHIT project’s telehealth support offer to a group of physicians from the practice who could disseminate the information to the remaining staff members and agreed on the methods. A web-based schedule was organized and hosted on the practice’s Google workspace, where the primary care physicians could schedule the telehealth session with the cardiologists.

In a preliminary meeting, all participants were previously trained in telehealth by one of the researchers (LG). In total, 1 to 3 cases of patients with heart failure were discussed in each session, which could take place once a week unless there was no appointment. The primary care physicians used the practice’s computers, and the cardiologists used the INC research department computers to connect and interact via the Zoom platform (Zoom Video Communications) licensed for the project. Phase A lasted from August 2020 to June 2021 (11 months).

#### Phase B: Asynchronous Telehealth Using an Online Platform

**Figure 1:**
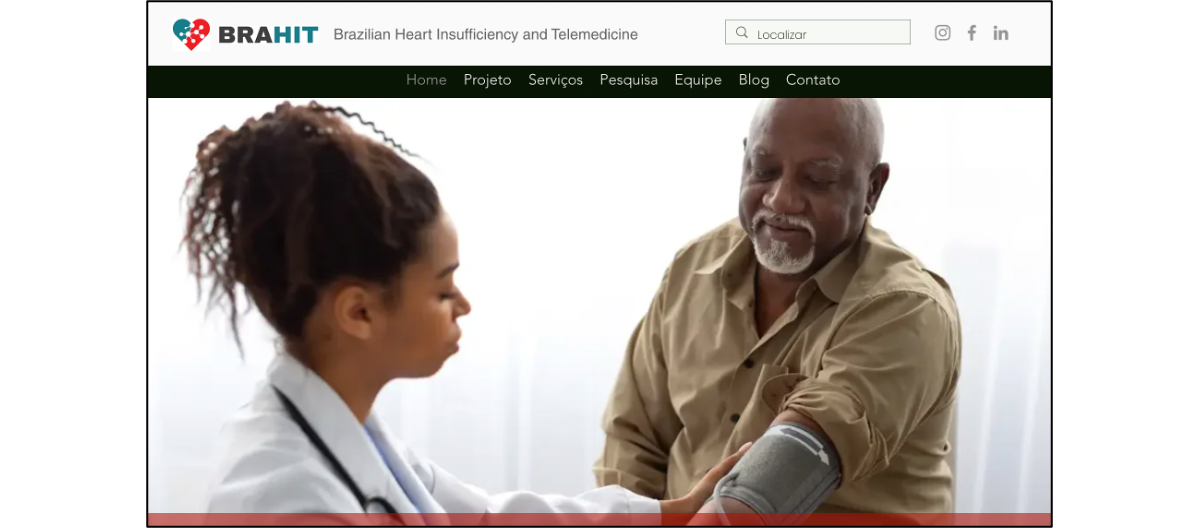
Telehealth online platform landing page used in all study phases for intervention delivery (provider-to-provider support from cardiologists to primary care physicians via telehealth) from August 2020 to December 2022. Permission obtained by the authorship for the use of the image without attribution.

Phase B started in July 2021, when the researchers decided to upscale the telehealth offer to all other primary care practices in the city. An IT company was hired to develop an online platform conceived by the researchers and based on similar experiences described in the literature [60] to allow for information exchange via text (asynchronous), substituting videoconferences as the initial interaction tool. The web-based platform was hosted on the project’s website (Figure 1).

**Figure 2:**
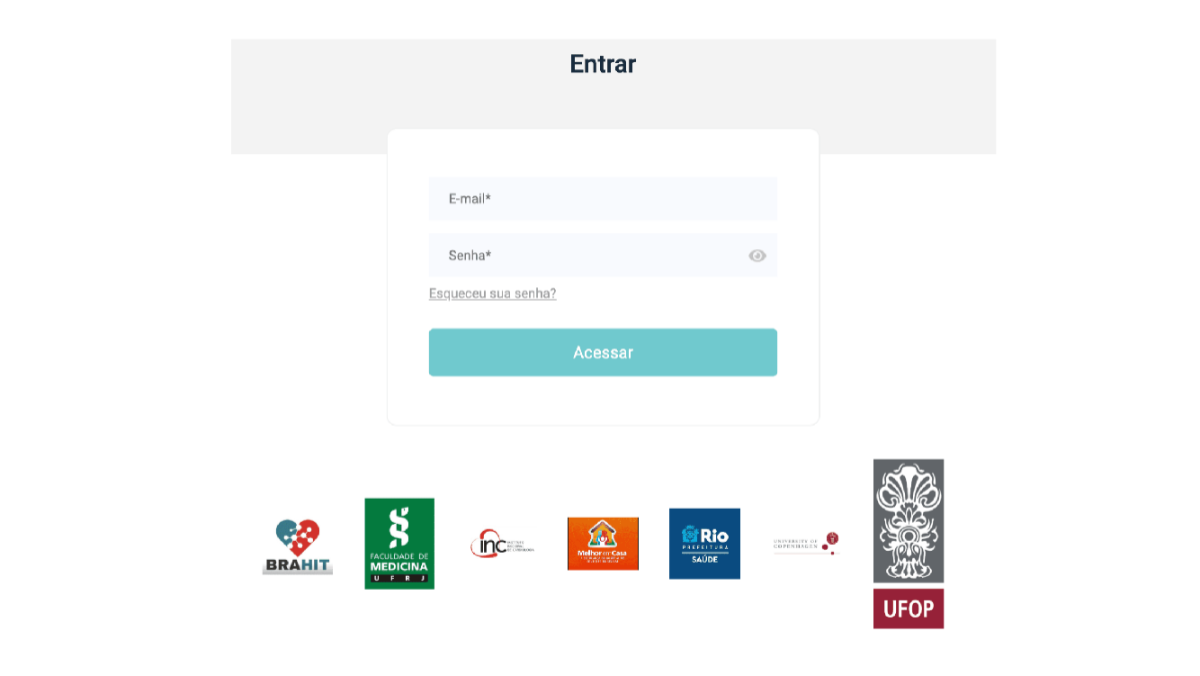
Log-in page for the online platform, restricted to registered users to protect data access and ensure their safety.

Upon registration and secure access granted by the research data management team (Figure 2), the primary care physicians entered their professional identification and contact information, the patient’s demographic and clinical data, and the reason for telehealth.

The research group’s teleconsultant should respond within 2 working days through a texting service within the platform. If primary care physicians deemed it necessary, they could still make synchronized phone or videoconference calls on demand. In this case, after agreeing with the cardiologist, they would use the WhatsApp app (Meta Platforms) for voice or video calls at their discretion. The web-based platform did not offer synchronous contact in the form of audio or video calls due to time and financial constraints for the tool’s development.

One of the researchers (LG) shared the BRAHIT project’s telehealth offer through presentations to the municipal health department, the regional primary care health coordination offices, and the family medicine residency program staff. In this second phase, 13 primary care practices participated in the telehealth program, including the practice involved in phase A. While primary care physicians could discuss cases of patients with other cardiologic diagnoses, this study focused solely on the discussion of heart failure cases.

In both phases, the duration of support was at the discretion of the primary care physicians. Regardless of the study phase, all patients had access to standard care, including consultations with physicians and nurses, preventive measures, oral health treatments, and follow-up visits from community agents. Participating primary care teams received weight scales, automatic blood pressure monitors, and oximeters to encourage patient follow-up. Phase B lasted from July 2021 to December 2022 (19 months).

### Participants and Data Collection

#### Qualitative Methods

We conducted 3 separate focus groups (group 1, group 2, and group 3) after the end of phase A and 15 interviews after phase B. The first author, LG, a physician and PhD candidate, scheduled, organized, and conducted the focus group sessions, whereas PCM, a female physician and master’s degree candidate, conducted the individual interviews. Both are trained in executing qualitative research data collection. MKG, a female researcher with robust qualitative research experience, supervised and supported data collection and analysis.

At the beginning of all focus group sessions, LG explained the research and session objectives and disclaimed the research objectives and premises, including the group’s assumptions and theories. Probing questions were used as an orientation for each focus group to facilitate the meeting interactions. All meetings were audio recorded for later transcription and content analysis. The probing questions of the semistructured interview script were about telehealth within the BRAHIT project, its use in the practices, and participants’ perception of their ability to manage patients with heart failure.

For group 1, researchers MKG and LG invited all the primary care physicians from the phase A practice, including family and community medicine specialists or residents. Considering the initial response of 5 family physicians and 10 residents, the researchers decided to conduct 1 session because a second one could have low attendance due to the participants’ time constraints. All invitees attended the session. With one exception, most participants were young physicians who had graduated in the previous 10 years. They are an engaged, proactive health care team that is usually cooperative and prone to quality improvement initiatives. All primary care physicians using telehealth and participating in this focus group were members of the Rio de Janeiro municipality’s family medicine residency program. This could have contributed to better engagement and assessment of educational activities such as telehealth. One of the primary care physicians was assigned as the observer. The session, which lasted 96 minutes, took place on June 22, 2021, in the practice auditorium.

For group 2, all 5 cardiologists who provided telehealth support during the study were considered eligible for the session and invited. The cardiologists have a strong connection with the researchers and vice versa as they are also project workers or researchers. In total, 80% (4/5) of the invited cardiologists attended the focus group session. One could not be contacted and had already left the project team. The senior author (HD) participated as an observer. The age range of the group was 31 to 54 years. A total of 50% (2/4) of the participants were male, and 50% (2/4) were female. Their cardiology practice time ranged from 3 to 32 years. The session was held through videoconference using the Zoom software on June 30, 2021, and lasted 90 minutes.

For group 3, we considered eligible the 32 patients whose cases were discussed during the videoconference sessions. Unfortunately, half (16/32, 50%) of them could not be contacted due to communication hardships or other unspecified reasons. The researchers relied on the help of the community health workers from the practice for invitations. LG and MKG invited all 16 contactable patients and decided to program 1 session, forecasting a nonattendance rate of at least 30%. In total, 31% (5/16) of the invited patients and the daughter of 1 patient, who was also his caregiver, attended the meeting on July 21, 2021, at the practice’s auditorium. The caregiver also contributed to the content but was identified as a patient due to privacy measures. The meeting lasted 63 minutes and was supervised by MKG, with 1 primary care physician as an observer.

For the individual interviews during phase B, we considered all 19 primary care physicians who worked as chief physicians of their respective practices in a different city region from that of the primary care practice in phase A. All accepted the invitation. A total of 79% (15/19) were women, and 84% (16/19) were White. The years of experience in primary care varied from 3 to 15 years. The interviews were conducted at the participants’ workplace in the practice’s lounge during work hours at a previously scheduled date and time. Importantly, medical staff and resource shortages were frequent in this region, especially during the COVID-19 pandemic, which coincided with the study period. This may have contributed to different attitudes and points of view regarding the same intervention. The interviews took place in December 2022.

The sampling for the qualitative methods was purposefully determined. The participants were considered to adequately represent the study populations as they were directly (primary care physicians and cardiologists) or indirectly (patients) involved in the telehealth process. The assessment of data saturation for the focus groups could not be planned because, despite previous consideration of repeating sessions with further participants, time constraints precluded more focus group sessions. The individual interviews had a high attendance rate (19/19, 100%), so the proposed sample was reached and considered representative of the studied population. To ensure trustworthiness, the data content from each focus group session and interview was primarily assessed as satisfactory by at least 2 researchers (MKG, LG, or PCM) at the end of each data collection activity. Due to operational reasons, transcriptions were not returned to the participants for feedback.

Data were recorded using the embedded audio recorder from LG’s cellphone (iPhone SE [Apple Inc]) for the focus groups and the Telegram app (Telegram FZ-LLC) on PCM’s phone for the individual interviews. All content was transcribed using the Transkriptor online platform [61] and stored locally on the investigators’ PCs (LG or PCM, respectively, for the focus groups and interviews) with no online access.

#### Quantitative Methods

In both phases of the project, we included all patients with heart failure whose cases were discussed in a telehealth session in the study. We excluded patients initially selected by the primary care physicians whose cases were not addressed in telehealth sessions. The sample size was not calculated for the quantitative assessment as hypothesis testing was not intended [56,62]. Therefore, we analyzed the baseline data of all the included participants in the study and the data after the intervention when there were enough data to be analyzed.

#### Quantitative Data

The primary care physicians registered the clinical data from the case discussions on electronic health records. For research purposes, the teleconsultants also entered data from the telehealth sessions on a REDCap (Research Electronic Data Capture; Vanderbilt University) database [63] hosted on a secure server at the INC and accessible only to the research team. The Rio de Janeiro municipality health department granted remote access to the electronic health records to follow up on the patients.

### Data Analysis

#### Qualitative

The transcripts were imported to the NVivo software (version 12 for transcripts from group 1 and 2 sessions and version 14 for individual interviews with physicians; Lumivero). The software version changed over the study period due to a change in license permissions by one of the research institutions [64]. MKG, LG, and PCM double-checked the content for transcription accuracy and corrected occasional mistakes in the electronically transcribed content to ensure the accuracy and confirmability of the dataset. To ensure the participants’ anonymity, we identified the content by the letter corresponding to the group. We attributed *C* to cardiologists, *FP* to family physicians, *P* to patients, and *IP* to individually interviewed physicians followed by a numeral according to the order of answers within the group. We did not add notes to capture nonverbal information.

In total, 3 researchers (LG, MKG, and PCM) analyzed the transcripts using thematic analysis as the primary approach [50,65-67]. First, the authors performed a general collective reading, obtaining first impressions about the content. They then explored the content, breaking it down into sentences (units). The units were coded initially as subthemes and then classified into broader themes. The coding proceeded dynamically during the reading, driven by the content, the guiding questions, and the authors’ perspectives. It was cyclical, involving rereadings until all sentences were classified. Repetitive statements were discarded. The 3 authors involved in data analysis worked together in 4 weekly in-person sessions using member checking and triangulation to enhance the analysis’s credibility and dependability.

Finally, the information was summarized, enabling the critical analysis of the material from the authors’ perspective. The authors emphasized the inductive interpretation of the content [65], analyzing the participants’ points of view and stories rather than quantitative variables such as the frequency of themes or codes.

LG, MKG, and PCM had in-person discussions to execute the data analysis and interpretation until they reached a satisfactory consensus considering different opinions and interpretations. The contents of each focus group session and the interviews were analyzed separately.

LG, MKG, and PCM had previous professional relationships with participants in the focus groups and individual interviews. LG was the former primary care coordinator in Rio de Janeiro and had previously collaborated academically with the involved cardiologists. MKG is an associate professor at the university who runs the internship program at the primary care practice from study phase A. PCM was the medical coordinator of the group of individually interviewed primary care physicians during the study period. These factors bring critical reflexivity to the data collection and analysis as the authors are linked to the health services they study and have personal intents and assumptions regarding assessing the study intervention, for example, the expectation of positive outcomes.

#### Quantitative

We collected data on demography (age, sex, and race), anthropometry (weight and BMI), vital signs (blood pressure and heart rate), heart failure decompensation (defined as the presence of pulmonary rales, jugular vein stasis, or leg edema on examination), and prescribed drugs and dosage. To assess GDMT in patients with HFrEF, we considered the 3-drug regimen of renin-angiotensin-aldosterone system inhibitors (RAAS-I), β-blockers, and mineralocorticoid receptor antagonists. We observed whether the drugs were used and the target doses were reached [68]. As we collected data from 2020 to 2022, when the recommendation of sodium-glucose cotransporter 2 (SGLT-2) inhibitors in guidelines as the fourth treatment *pillar* [22,69] was not yet consolidated in medical practice or incorporated into local guidelines [68], we decided not to consider the prescription of this drug class in our assessment of GDMT. Therefore, the use of SGLT-2 inhibitors was registered but not included in the GDMT analysis.

We analyzed the data using simple descriptive statistics. We described the baseline variables of all included patients. For the subgroup of patients with follow-up data, we described and compared the proportion of patients who were decompensated. Among those, we compared the proportion of patients with HFrEF who used GDMT.

All comparisons were between baseline and the latest time point within the year after the intervention, grouped by phase. Inferential statistics were not executed because the study objective was not to test any hypothesis based on the study data. If there was more than one measurement for the same patient during follow-up, we considered only the latest time point value.

### Outcomes

The primary outcome was the feasibility of telehealth support. To draw inferences about this outcome, we integrated the qualitative exploratory findings of the content analysis of the focus groups and individual interviews with quantitative data such as patients’ baseline data, clinical status, and the primary care physicians’ use of GDMT. For data integration, we connected the data within selected feasibility domains described by Aschbrenner et al [70] (eg, recruitment capacity, assessment procedures, implementation resources, intervention delivery, and acceptability). For decisions about feasibility and progression to the main trial, we used the A Process for Decision-Making After Pilot and Feasibility Trials framework for feasibility analysis described by Bugge et al [71]. We presented the integration results in the form of a joint display [72].

### Ethical Considerations

This study was carried out following the Declaration of Helsinki and approved by the INC (registration 5272), the health department of the Rio de Janeiro municipality (registration 5279), the Federal University of Ouro Preto (registration 5150), and the Brazilian National Research Ethics Committee (registration 8000) under application 14894819.5.0000.5272. The assessment by the Danish Research Ethics Committee System was waived because the study did not involve Danish participants or the use of Danish data.

Patients and primary care physicians involved in the study were informed and included only after signing informed consent forms tailored to each participant category. These forms served as a formal invitation to the study explaining the rationale behind the research and detailing characteristics such as the number of participants and the study duration. We also outlined the proposed activities and disclosed the potential benefits and risks of participation. Additional topics included information on data handling and use, confidentiality, and privacy, along with clarification about involvement in the study and the absence of financial or other forms of compensation for participation.

Regarding data collection and use, the researchers sought access from the local health authority to private demographic and clinical data available in the primary care health services’ electronic health record system (VitaCare). The Rio de Janeiro municipality granted authorization after we signed a statement of responsibility for data use. The informed consent permits secondary analysis without requiring additional permission.

The research team monitored patient data throughout the study. To ensure data safety, only 1 researcher and 2 undergraduate students had access to extract data from the electronic health records and input them into the study’s REDCap databases. The data were pseudoanonymized, with participants identified by their national health registration numbers. The REDCap database was subsequently made available to the rest of the research team in Brazil. Case management remained unaffected except for the eventual modifications in medical decisions influenced by telehealth. All procedures adhered to relevant laws and institutional guidelines.

### Registration

The BRAHIT frame project is registered at ClinicalTrials.gov under the number NCT04466852 and was approved by Brazil’s National Research Ethics Committee under the registration number 14894819.5.0000.5272.

### Procedural Diagram

**Figure 3:**
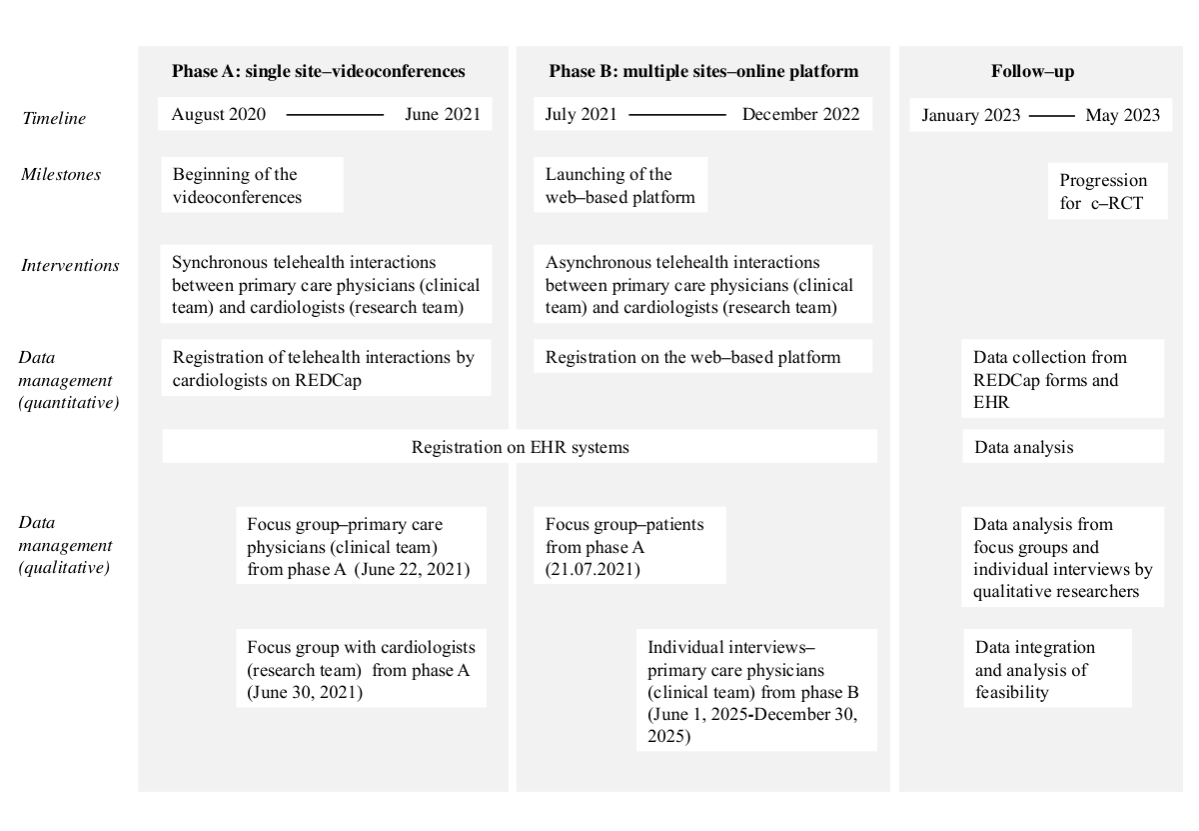
Procedural diagram—timeline, interventions, tasks, and data management by study phase. c-RCT: cluster-randomized controlled trial; EHR: electronic health record; REDCap: Research Electronic Data Capture.

In Figure 3, we present a procedural diagram [55] containing the timeline, the researchers’ tasks, participant activities, and data collection methods according to each project phase to ensure clarity in the study methods and execution.

## Results

### Qualitative Results

#### Common Findings

The content of all qualitative activities had telehealth support as a common theme due to the specific probing questions posed to all participants. Conversely, particular themes emerged based on the participant categories. For instance, concerns about patients’ social conditions and interactions among health care sectors were highlighted among primary care physicians in phase A but were less evident among those in phase B, where the themes focused more on professional matters. Differences in physicians’ educational backgrounds may explain this variation. All primary care physicians in phase A (focus group; 15/15, 100%) specialized in family and community medicine, whereas only 37% (7/19) in phase B (individual interviews) had the same specialization.

On the other hand, the physicians interviewed in the project’s phase B were more experienced than the ones in phase A. Different data collection methods (interview vs focus group) could have also played a role. In the case of the cardiologists, the operational aspects were notably frequent, which correlates with the fact that they were the consultants and research team members. In the patient focus group, the themes actively mentioned by the participants were related to the primary care service organization and their experience with disease and care. Each group’s code classification, findings, and interpretation are detailed in the following sections.

#### Focus Group: Primary Care Physicians

##### Overview

Four themes emerged from the session’s content analysis: (1) population aspects, (2) clinical competence in primary care, (3) communication among health care services, and (4) telehealth support. The themes, subthemes, and definitions are shown in Table 1.

**Table 1:** Focus group 1 (primary care physicians)—themes, subthemes, and definitions that emerged from content analysis.

Theme and subtheme			Definition
**Population aspects**			
	Disparities	Opinion on the population’s socioeconomic and cultural vulnerability	
	Mobility	Patients’ mobility hardships	
**Clinical competence in primary care**			
	Confidence	Lack of confidence in managing patients with heart failure	
	Task perception	Perception of the task of treating patients with heart failure	
Communication among health care services			Communication gap among health care sectors
**Telehealth support**			
	Use	Discussion about the use of supporting tools	
	Potential and barriers	Assessment of telehealth support use	

##### Population Aspects

Considering the context in which the focus group took place, a socially deprived area of the city, and the educational background of the participants, who were trained to deliver person-centered, community-oriented care, the mention of social disparities and their impact on patient care and the service organization was expected. The discussion highlighted the population’s socioeconomic and cultural vulnerability, which markedly influences their lives and clinical follow-up [73]:

...our patients are very vulnerable...So economically, intellectually, and culturally speaking, they need us.

FP1

Another important subtopic was *mobility*, reflecting the concerns of the primary care physicians about the patients’ itinerary within and between health care services. The patients’ difficulties moving around the city for an eventual referral to a specialized service were reported, reinforcing the importance of the primary care practice offering close, accessible, and comprehensive care, facilitating adherence. This aspect is supported by findings from the literature correlating the accessibility of primary care facilities and its impact on the continuity and quality of primary care delivery [74,75]:

...They don’t have the financial conditions to do it (commuting) from their pocket. So, they will return to us to continue care.

FP1

##### Clinical Competence in Primary Care

An essential theme that emerged from this focus group was the primary care physicians’ confidence in assisting patients with cardiologic conditions such as heart failure. The lack of confidence reported by some physicians regarding themselves and their colleagues may be due to inexperience and insufficient training before graduation:

...We know some topics more basically, like reading an X-ray or an electrocardiogram. I think the EKG is a general difficulty.

FP3

There was also sometimes a notably unclear perception of primary care as a scenario for managing severe diseases such as heart failure:

...I always imagined that I would manage...here in primary care, only hypertension, so anything that goes a little beyond within cardiology topics, literally, I don’t know.

FP2

##### Communication Among Health Care Services

When collaborative care is discussed, one main topic that usually emerges is the communication hardships between services [76]. The participants described significant communication problems, which led to gaps and unawareness of actions performed in secondary and tertiary services, affecting the patients’ care:

...I think the great difficulty we have today is that we seldom receive a report from a specialist. They should tell us how shared care is supposed to happen...

FP1

Sometimes, they order tests or prescribe medication, and we don’t know exactly why. How can I share the care with them and continue if I don’t know where they want to go?

FP3

##### Telehealth Support

The researchers’ questions probed the ubiquitous theme of teleconsulting services. The group discussed the ideal characteristics of a teleconsulting service, their experience with the BRAHIT project, and other support activities. The group evaluated telehealth support positively as it was easily accessible. They also assessed the BRAHIT project as having favorable characteristics:

...the intimacy, the ability (of the teleconsultants) to understand my difficulty, because sometimes I ask a question, and he already answers...

FP9

...They are focal specialists who understand my reality and see that they are contributing not only to me, but to patient care.

FP5

On the other hand, the time-consuming effort required to be physically present during the videoconferences was a frequent negative feedback. This information led the researchers to refine the intervention, adapting the telehealth offer to include an asynchronous approach commonly used in other telehealth services [77]:

...We know that we are privileged, because there are a lot of physicians here, but in other clinics I have worked, I would rarely have the time to be online in a web conference.

FP10

#### Focus Group: Cardiologists

##### Overview

Two themes emerged from the session’s content analysis: (1) the relationship with the primary care service and (2) telehealth support. The themes, subthemes, and definitions are shown in Table 2.

**Table 2:** Focus group 2 (cardiologists)—themes, subthemes, and definitions that emerged from content analysis.

Theme and subtheme			Definition
**Relationship with the primary care service**			
	Vision on primary care	Discussion about their vision on primary care services	
	Mission	The National Institute of Cardiology’s mission as a teaching institution	
**Telehealth support**			
	Education	Evaluation of the interactions regarding collaboration	
	Challenges	Challenges of telehealth implementation	

##### Relationship With the Primary Care Service

The cardiologists discussed their preconception about primary care services, initially evaluated as deficient in structure and quality of human resources, and stated a paradigm shift after contact with the team from the primary care practice:

...we are hospitalists, and sometimes we believe that the primary care practice has an inadequate structure, right?

C1

Sometimes, physicians do not have adequate training, and it was a paradigm that was broken about the technical level of the colleagues, which is, in fact, very high.

C2

Another important finding was the recognition by the cardiologists of significant opportunities for the INC team, highlighting their role as a specialized public institution in education to improve the overall quality of the health care system:

...I noticed since the first time the chance not only to improve the follow-up of these patients but also to teach the professionals who work there, allowing them to feel more capable of helping people. I think that most people in primary care have this vocation.

C1

##### Telehealth Support

The telehealth interactions were assessed as positive regarding training and collaboration between the parties, and opportunities for bilateral learning were identified:

They already have a different perception of approaching cardiac patients, and it has been a very enriching exchange of experiences for both sides. Sometimes, I think we also learn from them.

C2

So, bringing not only knowledge but also the experience that we have in terms of treatment, I think general practitioners have good experiences with us and realize that we are calm. The patient is severe, but we manage it.

C4

The cardiologists reported concerns about implementing telehealth, specifically about its scalability and sustainability and the engagement of primary care physicians:

...I just think there was also an underuse of the service. I think it could have been used more.

C2

#### Focus Group: Patients

##### Overview

Two themes emerged from the session’s content analysis: (1) disease and care experience and (2) telehealth support. The themes, subthemes, and definitions are shown in Table 3.

**Table 3:** Focus group 3 (patients—phase A)—themes, subthemes, and definitions that emerged from content analysis.

Theme and subtheme		Definition
**Disease and care experience**		
	Health literacy	Understanding regarding their disease and care
	Insights about self-care	Thoughts about good habits and well-being
	Care evaluation	Assessment of physicians’ actions and consequences for their health
	Free will	Attitudes toward the disease
**Telehealth support**		
	Opinions and fears	Opinions and worries about telehealth support

##### Disease and Care Experience

The probing questions for the patients investigated their understanding of heart failure as a disease and their conceptions of medical assistance. Their discussions revealed a heterogeneous understanding of cardiologic conditions and their treatment:

...I used to think there was one type of heart disease. One would feel chest pain. But it seems that there is more than that. I do not understand.

P3

There were also reports about the patients’ improvements after they were properly diagnosed and treated. They could find a positive correlation between following correct habits and taking correct medications and their well-being:

...Then I do not feel tired anymore. It has been two years now. I cycle to work and to everywhere around. I help a friend with construction work. It is impressive. I even get suspicious sometimes.

P6

Nevertheless, in the words of other participants, we recognized a disconnection between their interpretation of physicians’ actions, test results, and medications and their feelings. We also noticed different attitudes toward the disease depending on individual characteristics:

...I only go to hospitals or clinics if I am dying. If I feel something that can be managed with analgesics or something, I will not come. I do not take prescription medications every day, as I feel myself controlled.

P4

##### Telehealth Support

The participants responded positively when discussing cardiologists’ telehealth support for their primary care physicians. They understood the initiative as an improvement. One participant reported that his physician participated in the BRAHIT project:

...He [the physician] takes pictures of the test results and sends them to the project. Yes, I think he is participating. Maybe it is working!

P6

...I think it is a very good idea.

P2

The literature does not extensively address the patient vision of telehealth between health care professionals. Our findings are significant as they provide the patients’ perspective on the strategy. In our findings, the patients seen in specialized care reported feeling unsafe enough to stop regularly attending specialist appointments even after the implementation of telehealth support:

...I think it would be better if we went to the hospital and had all the tests. It would be better to go directly there. Because it is a specialist.

P3

...I go to the hospital every three months. I feel safer going there, too.

P5

#### Individual Interviews

##### Overview

Four themes emerged from the interview content analysis: (1) work overload, (2) telehealth use, (3) clinical competence, and (4) referral practices. The themes, subthemes, and definitions are shown in Table 4.

**Table 4:** Individual interviews (primary care physicians—phase B)—themes, subthemes, and definitions that emerged from content analysis.

Theme and subtheme			Definition
Work overload			Influence of work rhythm on telehealth use
**Telehealth support**			
	Actual use	Experiences using telehealth	
	Barriers	Reasons for not using telehealth	
Clinical competence			Confidence in assisting patients with heart failure
Referral practices			Influence of telehealth in referring patients to specialists

##### Work Overload

Professionals usually describe the work context in Brazil’s primary care practices as being in high demand. Most practices have a high panel size, and the teams usually must deal with acute and programmed care. The scenario during our research was influenced by the COVID-19 pandemic, bringing further pressure to the practices and the political scene, where the Rio de Janeiro municipality was adopting an austerity policy, including staff reduction, which also played a role [78-80]. Therefore, the principal issue reported by the participants was the lack of available time due to an overwhelming burden of tasks and consultations:

We did not use the telehealth support because of the work overload in our practice, a significant physician shortage, and turnover. This jeopardized the dissemination and utilization of the tool.

IP2

##### Telehealth Support

Some participants reported a favorable experience and advantages, such as greater confidence in managing patients with heart failure and fewer referrals. They recognized the initiative’s potential for quality improvement:

...discussing cases of patients with heart failure with multimorbidity and decompensated cases provided greater confidence in managing the case and could reduce referrals to emergencies and specialists.

IP1

Conversely, cardiologists sometimes took a long time to respond to contact requests, which was considered a problem:

When I tried to use the website, connecting was hard. I found it slow. As other tools are available online, I do not use them anymore.

IP3

##### Clinical Competence

When asked about their ability and confidence in assisting patients with heart failure, most physicians answered that they could help. This finding brings about an interesting paradox because our quantitative data showed a poor clinical baseline status of most patients whose cases were discussed in the project:

...no need for questioning in cardiology; therefore, I have not used the telehealth support from the BRAHIT project. It is worth mentioning that we have a WhatsApp group for case discussions provided by the municipality health department.

IP5

Other reports mentioned a lack of interest, use of alternative tools, or no need to use telehealth support:

...in my population, there are no patients with heart failure needing specialist consultation, nor do I need telehealth support for myself.

IP6

##### Referral Practices

The traditional approach to treating complex cases in primary care involves referring patients to specialized services. A total of 16% (3/19) of the participants alleged that referring the patient to the cardiology service would be easier. Nevertheless, this approach may entail problems, such as low patient attendance due to the issues described previously, such as commuting difficulties, which are also reported in the literature [5,81,82]:

...When I need to refer the patient to a cardiologist, I use the referral system. So, the telehealth support offer and objectives are still not clear to me.

IP7

...The patients have already been managed via referral through the referral system.

IP8

### Quantitative Results

#### Participants

**Figure 4:**
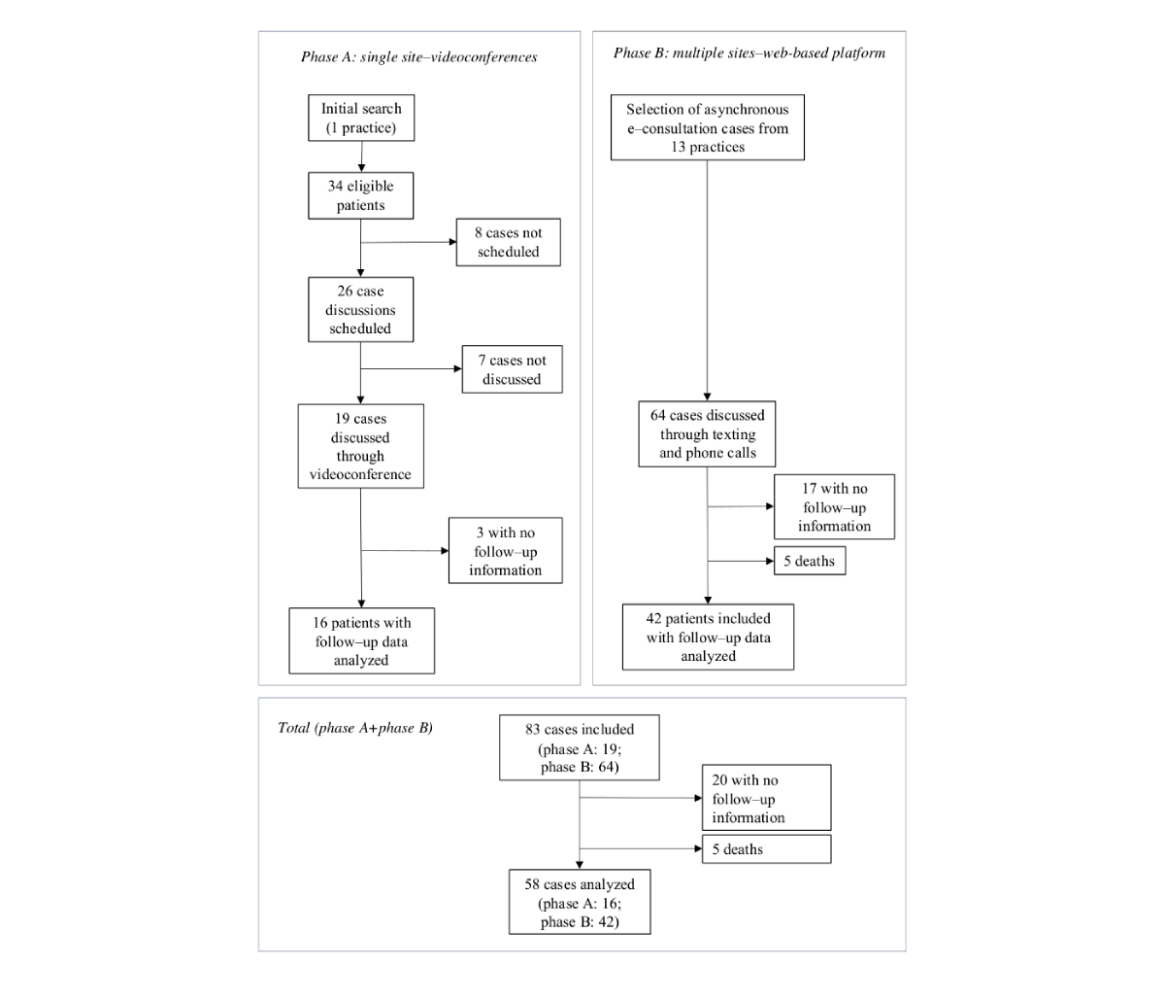
Flow diagram of patient inclusion in the study and quantitative before-and-after follow-up for 1 year based on the CONSORT (Consolidated Standards of Reporting Trials) framework for reporting clinical trials (data from August 2020 to December 2022).

During the videoconference phase (phase A) of the intervention, the physicians selected 34 patient cases for discussion, of which 26 (76%) were scheduled for discussion based on the physicians’ criteria and their availability to attend the telehealth session. A total of 27% (7/26) of these cases were not discussed for unknown reasons. In total, 73% (19/26) of the cases were discussed via videoconference. Follow-up data were available from the practice’s electronic health records for 84% (16/19) of these patients. In phase B, 64 patients from 13 primary care practices had their cases discussed asynchronously. Of these 64 patients, 5 (8%) died, 17 (27%) did not have further consultation records, and the remaining 42 (66%) were followed up on. Adding both phases, 83 cases were discussed, and 58 (70%) patients were followed up on. Participant inclusion is summarized in the flowchart in Figure 4.

#### Baseline Data

Regarding demographic data, the mean patient age was 61 (SD 12) years. Of the 83 patients, 52 (63%) were male, and 31 (37%) were female; of 73 patients with available data, 30 (41%) were White, and 28 (38%) were Black or belonged to another ethnic minority group. The proportion of common diagnoses associated with heart failure was similar to that in the literature except for chronic obstructive pulmonary disease, which was reported in only 2% (1/61) of the participants with available data, suggesting underdiagnosis [83]. Regarding anthropometry and vital signs, BMI and mean blood pressure and heart rate values were above the recommended limits. Of the patients with available data, 64% (7/11) in phase A and 45% (21/47) in phase B had HFrEF. Most patients (39/74, 53%) had poor physical status according to the New York Heart Association classification. The data are described in detail in Table 5.

**Table 5:** Baseline demographic and clinical data of all patients included in the quantitative assessment of this study (N=83).

Variable		Phase A (n=19)		Phase B (n=64)		Total
Age (y), mean (SD; range)		58 (12; 35-76)		61 (13; 37-89)		61 (13; 35-89)
**Sex, n (%)**						
	Female	7 (37)	24 (37)		31 (37)	
	Male	12 (63)	40 (63)		52 (63)	
**Race, n (%)**						
	Black or other ethnic minority group	6 (35)	22 (39)		28 (38)	
	White	9 (53)	21 (38)		30 (41)	
	Not informed	2 (12)	13 (23)		15 (21)	
	Missing	2 (11)	8 (12)		10 (12)	
**Atrial fibrillation, n (%)**						
	No	13 (76)	33 (70)		46 (72)	
	Yes	4 (24)	14 (30)		18 (28)	
	Missing	2 (11)	17 (27)		19 (23)	
**Diabetes, n (%)**						
	No	11 (65)	32 (62)		43 (62)	
	Yes	6 (35)	20 (38)		26 (38)	
	Missing	2 (11)	12 (19)		14 (17)	
**COPD^a^** **, n (%)**						
	No	13 (93)	47 (100)		60 (98)	
	Yes	1 (7)	0 (0)		1 (2)	
	Missing	5 (26)	17 (27)		22 (27)	
**Coronary artery disease, n (%)**						
	No	7 (88)	22 (49)		29 (55)	
	Yes	1 (12)	23 (51)		24 (45)	
	Missing	11 (58)	19 (30)		30 (36)	
**Hypertension, n (%)**						
	No	4 (21)	15 (25)		19 (24)	
	Yes	15 (79)	46 (75)		61 (76)	
	Missing	0 (0)	3 (5)		3 (4)	
**Stroke, n (%)**						
	No	15 (100)	56 (92)		71 (93)	
	Yes	0 (0)	5 (8)		5 (7)	
	Missing	4 (21)	3 (5)		7 (8)	
**Peripheral artery disease, n (%)**						
	No	15 (100)	56 (97)		71 (97)	
	Yes	0 (0)	2 (3)		2 (3)	
	Missing	4 (21)	6 (9)		10 (12)	
**Dyslipidemia, n (%)**						
	No	8 (62)	26 (53)		34 (55)	
	Yes	5 (38)	23 (47)		28 (45)	
	Missing	6 (32)	15 (23)		21 (25)	
BMI (kg/m^2^), mean (SD; range)		32 (7; 23-49)^b^		29 (6; 19-53)^b^		30 (6; 19-53)^c^
Systolic blood pressure (mm Hg), mean (SD; range)		138 (31; 97-220)		130 (29; 90-240)^b^		132 (29; 90-240)^b^
Diastolic blood pressure (mm Hg), mean (SD; range)		91 (21; 60-160)		80 (16; 40-120)^b^		82 (18; 40-160)^b^
Heart rate (bpm^d^), mean (SD; range)		81 (19; 53-125)^b^		79 (18; 42-121)^b^		79 (18; 42-125)^c^
**NYHA^e^** **functional classification, n (%)**						
	I	2 (12)	10 (17)		12 (16)	
	II	8 (50)	15 (26)		23 (31)	
	III	1 (6)	21 (36)		22 (30)	
	IV	5 (31)	12 (21)		17 (23)	
	Missing	3 (16)	6 (9)		9 (11)	
LVEF^f^ (%), mean (SD; range)		35 (8; 21-48)^g^		43 (19; 14-80)^h^		42 (18; 14-80)^i^
**Heart failure classification (LVEF status), n (%)**						
	Reduced	7 (64)	21 (45)		28 (48)	
	Mildly reduced	4 (36)	9 (19)		13 (22)	
	Preserved	0 (0)	17 (36)		17 (29)	
	Missing	8 (42)	17 (27)		25 (30)	
Creatinine (mg/dL), mean (SD; range)		1.3 (1; 0.7-5.1)^c^		1.3 (1; 0.6-8.0)^j^		1.3 (1; 0.6-8.0)^k^

^a^COPD: chronic obstructive pulmonary disease.

^b^Missing: n=1.

^c^Missing: n=2.

^d^bpm: beats per minute.

^e^NYHA: New York Heart Association.

^f^LVEF: left ventricular ejection fraction.

^g^Missing: n=8.

^h^Missing: n=17.

^i^Missing: n=25.

^j^Missing: n=3.

^k^Missing: n=5.

#### Outcome Analysis

We used data from 58 patients available in electronic health records within 1 year following the first telehealth interaction to assess changes before and after telehealth. The mean follow-up time after telehealth was 183 (SD 109; range 14-365) days. The proportion of missing data at follow-up was very high (mean 28%, SD 14%, varying from 1/21, 5% to 23/42, 55% depending on the variable), precluding a precise assessment or identification of patterns.

There was a modest change in the patients’ vital signs after follow-up compared to baseline. The mean systolic blood pressure was 7 mm Hg lower, the mean diastolic blood pressure was 3 mm Hg lower, and the mean heart rate was 3 beats per minute lower. The proportion of patients with signs of decompensated heart failure was 63% (17/27) compared to 50% (29/58) of patients at baseline. Of the patients with reduced ejection fraction assessed at baseline and during follow-up, 55% (12/22) and 55% (11/20), respectively, had prescriptions for the 3 main GDMT drug classes, which can be explained by an increase in β-blocker (17/20, 85% to 18/19, 95%) and RAAS-I (14/20, 70% to 15/19, 79%) prescription but a drop in the prescription of spironolactone (16/20, 80% to 15/20, 75%). Newer agents such as neprilysin and SGLT-2 inhibitors were introduced during the follow-up period for 4 and 1 patient, respectively, compared to no use record at baseline. The data are presented in detail in Table 6.

**Table 6:** Clinical data before and after telehealth support—subgroup of patients with at least one follow-up contact registered in primary care electronic health records (N=58).

Variable		Phase A (n=16)			Phase B (n=42)		Total	
		Before	After	Before		After	Before	After
Days between baseline and follow-up, mean (SD; range)		157 (109; 14-344)	—^a^	192 (99; 22-365)		—	183 (103; 14-365)	—
**Heart failure classification (LVEF^b^** **status), n/N (%)**								
	Reduced	7/9 (78)	7/9 (78)	15/31 (48)		15/31 (48)	22/40 (55)	22/40 (55)
	Mildly reduced	2/9 (22)	2/9 (22)	4/31 (13)		4/31 (13)	6/40 (15)	6/40 (15)
	Preserved	0/9 (0)	0/9 (0)	12/31 (39)		12/31 (39)	12/40 (30)	12/40 (30)
	Missing	7/16 (44)	7/16 (44)	11/42 (26)		11/42 (26)	18/58 (31)	18/58 (31)
Systolic blood pressure (mm Hg), mean (SD; range)		136 (33; 97-220)	134 (43; 90-260)^c^	132 (31; 90-240)		123 (22; 70-160)^d^	133 (32; 90-240)	126 (30; 70-260)^e^
Diastolic blood pressure (mm Hg), mean (SD; range)		91 (23; 60-160)	88 (21; 60-140)^c^	80 (17; 40-120)		77 (16; 40-109)^d^	83 (19; 40-160)	80 (18; 40-140)^e^
Heart rate (bpm^f^), mean (SD; range)		85 (19; 58-125)^g^	86 (20; 63-125)^h^	79 (18; 42-120)		74 (13; 43-100)^i^	80 (18; 42-125)^g^	77 (15; 43-125)^j^
**Signs of decompensated heart failures^k^** **, n/N (%)**								
	No	5/14 (36)	5/8 (62)	18/38 (47)		5/19 (26)	23/52 (44)	10/27 (37)
	Yes	9/14 (64)	3/8 (38)	20/38 (53)		14/19 (74)	29/52 (56)	17/27 (63)
	Missing	2/16 (12)	8/16 (50)	4/42 (10)		23/42 (55)	6/58 (10)	31/58 (53)
**GDMT^l^** **in HFrEF^m,n^** **, n/N (%)**								
	No	4/7 (57)	3/7 (43)	6/15 (40)		6/13 (46)	10/22 (45)	9/20 (45)
	Yes	3/7 (43)	4/7 (57)	9/15 (60)		7/13 (54)	12/22 (55)	11/20 (55)
	Missing	0/7 (0)	0/7 (0)	0/15 (0)		2/15 (13)	0/22 (0)	2/22 (9)
**β-blocker use in HFrEF, n/N (%)**								
	No	2/7 (29)	0/7 (0)	1/13 (8)		1/12 (8)	3/20 (15)	1/19 (5)
	Yes	5/7 (71)	7/7 (100)	12/13 (92)		11/12 (92)	17/20 (85)	18/19 (95)
	Missing	0/7 (0)	4/11 (36)	0/13 (0)		4/16 (25)	0/20 (0)	8/27 (30)
**MRA^o^** **use in HFrEF, n/N (%)**								
	No	3/7 (43)	3/8 (38)	1/13 (8)		2/12 (17)	4/20 (20)	5/20 (25)
	Yes	4/7 (57)	5/8 (62	12/13 (92)		10/12 (83)	16/20 (80)	15/20 (75)
	Missing	1/8 (12)	4/12 (33)	0/13 (0)		3/15 (20)	1/21 (5)	7/27 (26)
**RAAS-I^p^** **use in HFrEF, n/N (%)**								
	No	3/7 (43)	1/7 (14)	3/13 (23)		3/12 (25)	6/20 (30)	4/19 (21)
	Yes	4/7 (57)	6/7 (86)	10/13 (77		9/12 (75)	14/20 (70)	15/19 (79)
	Missing	0/7 (0)	4/11 (36)	0/13 (0)		2/14 (14)	0/20 (0)	6/25 (24)
**Neprilysin inhibitor use in HFrEF, n/N (%)**								
	No	7/7 (100)	5/8 (62)	13/13 (100)		11/12 (92)	20/20 (100)	16/20 (80)
	Yes	0/7 (0)	3/8 (38)	0/13 (0)		1/12 (8)	0/20 (0)	4/20 (20)
	Missing	2/9 (22)	5/13 (38)	0/13 (0)		3/15 (20)	2/22 (9)	8/28 (29)
**SGLT-2^q^** **inhibitor use in HFrEF, n (%)**								
	No	7/7 (100)	7/8 (88)	12/12 (100)		12/12 (100)	19/19 (100)	19/20 (95)
	Yes	0/7 (0)	1/8 (12)	0/12 (0)		0/12 (0)	0/19 (0)	1/20 (5)
	Missing	2/9 (22)	5/13 (38)	1/13 (8)		3/15 (20)	3/22 (14)	8/28 (29)

^a^Not applicable.

^b^LVEF: left ventricular ejection fraction.

^c^Missing: n=5.

^d^Missing: n=13.

^e^Missing: n=18.

^f^bpm: beats per minute.

^g^Missing: n=1.

^h^Missing: n=7.

^i^Missing: n=19.

^j^Missing: n=26.

^k^Pulmonary rales, jugular stasis, or leg edema.

^l^GDMT: guideline-directed medical therapy.

^m^HFrEF: heart failure with reduced ejection fraction.

^n^GDMT—at least one renin-angiotensin-aldosterone system inhibitor+1 β-blocker+1 mineralocorticoid antagonist.

^o^MRA: mineralocorticoid receptor antagonist.

^p^RAAS-I: renin-angiotensin-aldosterone system inhibitor.

^q^SGLT-2: sodium-glucose cotransporter 2.

### Data Integration and Feasibility Assessment

The content analysis of the focus groups and individual interviews gave us a clear view of the intervention context, allowing us to identify some patterns. While assessing the feasibility of the intervention, we received critical feedback. We obtained significant insights on the implementation context and potential barriers and facilitators for the planned intervention to be appropriately delivered within the upcoming cluster-randomized trial. In turn, the quantitative analysis showed the baseline status regarding the patients’ demographics and clinical characteristics and some change tendencies in the primary care physicians’ prescription practices after telehealth implementation.

To draw inferences about both data types, we interconnected the main findings and correlated them with feasibility domains [70] when applicable. We concluded that the intervention is feasible, with adjustments, as described in the A Process for Decision-Making After Pilot and Feasibility Trials model items *adapting the intervention*, *adjusting the clinical context within which the intervention would be delivered*, and *amending elements of the trial design* [71]. Practically, during the feasibility trial, we decided to use the asynchronous telehealth method and recruit patients discharged from hospitals and emergency rooms in the future cluster-randomized trial instead of only including the patients selected by the primary care physicians. Table 7 consolidates the main findings, interpretations, and decisions regarding feasibility in a joint display.

**Table 7:** Joint display of results and mixed methods interpretations integrating qualitative and quantitative findings.

Domain	Quantitative results	Qualitative results	Mixed methods interpretation	ADePT^a^ actions
Setting	Of 73 patients with available data, 30 (41%) were White, and 28 (38%) were Black or from other ethnic minority groups, contrasting with the population of the study. The mean age of the study participants was 61 years, 4.5 years lower than the mean reported age in Brazil of patients with heart failure.	Primary care teams reported lack of physicians in individual interviews. The population covered by the practice is socioeconomically vulnerable and has insufficient knowledge about their condition and care.	The setting is challenging, requiring active involvement of all stakeholders. Facing difficulties, physicians may privilege patients with easier access to care. Actions integrated with telehealth support aimed at patient health literacy could be synergic.	Adapt the intervention for the setting conditions. Be aware of possible access hardships for non-White populations. Design cointerventions to overcome barriers (eg, patient education activities).
Recruitment capacity	A total of 83 patients had their cases discussed in 2 years in the practices where physicians used the telehealth offer. Only 1 in 15 physicians who participated in the individual interviews used the telehealth offer.	Lack of awareness on the part of the primary care physicians of their need for support. Work overload hindered the use of cardiologist support with telehealth.	The results agree and are likely to have a strong correlation. An active search by the research team of patients suitable for telehealth could help.	Modifying the intervention to include a nudging strategy for telehealth use would favor recruitment. A decision was made to include actively sought out postdischarge patients in the subsequent trial.
Assessment procedures	Identification of improvement opportunities from the baseline clinical data Use rate of newer agents to treat heart failure improved from 0 (0%) to 5 (20%). Lack of effect in other quantitative outcomes (eg, patients who were decompensated)	Both teleconsultant cardiologists and family physicians are optimistic about using telehealth as a tool for care improvement. Lack of awareness of support need by some primary care physicians related to the telehealth offer	The results agree and are likely to have a strong correlation.	The intervention is feasible and potentially beneficial for the clinical performance. Design cointerventions to overcome barriers (eg, professional education activities).
Intervention delivery	Identification of improvements related to the intervention Use rate of newer agents to treat heart failure improved from 0 (0%) to 5 (20%).	Positive feedback from the participants from the primary care teams Videoconferences were time-consuming.	The results agree and are likely to have a correlation.	The intervention is feasible if adapted. The intervention was modified for asynchronous communication in phase B.
Implementation resources	The upscaled offer of telehealth was rapidly accepted in 13 primary care practices in phase B. The telehealth offer seemed cost-effective and did not cause a burden to the project finances.	The feedback from teleconsultants was positive. The sustainability of the offer was a concern in the cardiologist focus group.	The results agree and are likely to have a correlation.	The intervention is feasible.
Acceptability	There was no refusal from primary care physicians to participate in the study, although compliance with the intervention was low in some settings.	Content analysis of the patient focus group revealed restrictions regarding the intervention as it could be a risk for prompt access to specialized care.	There was an attention point regarding the guarantee of access to specialized care.	The intervention can be tailored to include clarification about no access block for the patients.

^a^ADePT: A Process for Decision-Making After Pilot and Feasibility Trials.

## Discussion

### Principal Findings and Interpretation

In this study, we aimed to assess the feasibility of telehealth support from cardiologists to primary care physicians for the care of patients with heart failure in the community setting. We analyzed factors from the study context, stakeholders’ attitudes and perceptions, barriers, facilitators, and possible influence on clinical practice.

The content analysis from focus groups and individual interviews revealed a favorable opinion when participants were asked about telehealth. In parallel, aspects of the intervention’s context emerged, such as the population’s socioeconomic conditions and primary care professionals’ work environment, collaboration with other health care sectors, and professional educational background. Considering these aspects and others that may ensue in different contexts is vital while implementing and assessing telehealth interventions, as in any innovation strategy.

The assessment of context and human factors has been described as essential in several publications about social, complexity, and implementation science. Therefore, the findings of this feasibility study are consistent with the literature on complex interventions involving knowledge-seeking behavior, including eHealth technologies. In a review about spreading and scaling innovation and improvement, Greenhalgh and Papoutsi [42] add *develop adaptive capability in staff*, *attend to human relationships*, and *harness conflict productively* as principles to be followed when planning the change programs described by Lanham et al [84]. Other reviews and editorials by Robert et al [41], Greenhalgh et al [42,43], and Greenhalgh and Russell [85] refer to some hardships that we also found in our study.

Phase B participants who were interviewed reported low engagement and acceptance due to work overload. The findings echo some reports in the literature. One specific scoping review on shared decision-making strategies using digital health technology in cardiovascular care points to *increased work responsibilities* as the most frequently reported barrier [86]. The low perception of the relative advantage of telehealth, present in the analysis of individual interviews, can hinder the implementation of innovations and, therefore, must be addressed and discussed before the implementation of telehealth [87]. This finding contrasts with recent surveys about continuing medical education in primary care, where the most frequent reasons for low engagement, in addition to work overload, were the inability to use digital tools and the difficulty in integrating the process into the practice routine [88].

Another key finding was the patients’ preoccupation that telehealth support could block their access to specialized services. This points to the need to reassure the patients that access to the focal specialists will still be available when using telehealth. The literature does not usually describe the patients’ perspective on provider-to-provider telehealth. We believe that including their assessment is essential and highly recommended in feasibility studies [89].

Regarding demographic data, the patients’ mean age was 4.5 years lower than the Brazilian average reported by the National Brazilian Registry of Heart Failure [90]. We believe that the participants’ low socioeconomic status plays a role in this disparity. Studies show an earlier and higher exposure to suboptimal nutrition habits and low self-care in socially deprived populations, anticipating the development of risk factors and diseases that will cause heart failure [73,91]. There was also a low proportion of participants who were female, Black, and of other ethnic minority groups in this study, contrasting with the more frequent use of health care services by women [92] and the higher heart failure prevalence among Black people and those of other ethnic minority groups [93]. The demographic profile of our sample may indicate a selection bias by the primary care physicians when including the patients for case discussion. This finding is supported by other authors describing equity discrepancies and underrepresentation of minority groups regarding access to care [94] and research participation [95].

The quantitative analysis showed opportunities for improvement in patient care. At baseline, more than half (39/74, 53%) of the patients with available data had poor functional capacity. The low rate of GDMT use may be a reason as only 55% (12/22) of the patients with HFrEF had prescriptions according to the recommended local and international guidelines. Unfortunately, this phenomenon is frequently reported in the medical literature [8,25,69,96]. We evaluate the tendency toward GDMT as favorable, with increases in the use of all drug classes except spironolactone, whose prescription decreased. Possible reasons include variations in drug availability in primary care, as physicians usually prescribe what is available for the patients to collect for free in the practices, or the primary care physicians’ lack of familiarity with the drug. The Change the Management of Patients With Heart Failure registry published by Greene et al [24] showed that mineralocorticoids were the least prescribed drug among the 3 categories (not prescribed in 67% of the patients vs 27% and 33% of the patients not being prescribed RAAS-I and β-blockers, respectively). However, the small number of participants assessed for this outcome does not allow us to draw accurate conclusions.

Integrating qualitative and quantitative data allowed us to foresee elements to be tailored in the forthcoming clinical trial as we evaluated its context, stakeholders’ attitudes, and other practicalities. We deemed the feasibility analysis positive considering the adjustments and complementary strategies within the research’s reach. Accordingly, we changed the recruitment strategy, selecting patients discharged from hospitals and emergency rooms because of heart failure instead of depending on primary care physicians’ spontaneous use of telehealth. We also defined the asynchronous telehealth model as the intervention and planned the implementation of educational activities to engage the target stakeholders [46].

### Strengths

This study’s strength lies in its use of mixed methods to analyze data integration between the participants’ opinions and the possible changes caused by telehealth. Mixed methods are recommended for studying the feasibility of complex interventions such as telehealth [48]. Integrating qualitative and quantitative data allows for a more thorough description of the intervention’s development and provides specific answers for researchers, allowing for a better assessment of the feasibility domains [57,70]. Another strength was using a particular framework for decision-making in feasibility trials considering the context and human factors that hinder or facilitate the intervention.

This study took place in primary care practices in Rio de Janeiro, which is a rich environment for clinical research due to its large dimensions, organization, and systematic use of electronic health records [97]. Most studies about telehealth have been conducted in high-income countries [29]. Hence, our findings will likely be transferable within Brazil and other countries with similar socioeconomic conditions and health care systems. Finally, we included the patients’ vision on the intervention. Although provider-to-provider telehealth does not directly involve patients as participants, its ultimate goal is to improve their medical care. Patients’ assessment of provider-to-provider telehealth has been investigated in a few studies by some research groups from North America [39].

### Limitations

Our trial has several limitations. The first limitation related to the study design is using a concurrent mixed methods approach where quantitative and qualitative data are collected simultaneously. This decision was driven by time and operability constraints. Nevertheless, we believe that it did not significantly affect inferences or interpretations. We relied on reports from the literature stating that concurrent designs are frequently used in health care research due to their efficiency regarding time and data collection [98].

The second limitation is the occasional synchronous communication between the primary care physicians and cardiologists during phase B, such as WhatsApp texting and audio and video calls. Although it was a deviation from the planned intervention, we decided to keep it to ensure the study’s pragmatism. The interactions were not frequent, but we unfortunately did not track them as the measurement was not planned in our data collection strategy.

The third limitation is the sampling strategy for the focus groups. We had 1 focus group session with family medicine specialists and residents, 1 with patients from study phase A, and 1 with cardiologists. Of the 15 invited patients, only 5 (33%) attended the session, which could limit data availability. Therefore, a traditional data saturation assessment of the focus groups was not conducted as described in the literature [99]. Nevertheless, the researchers believe that the topics addressed in the focus groups covered most aspects of telehealth feasibility. In addition, participants mentioned other topics that enriched the content analysis. A review by Tausch and Menold [100] describes the advantages of “smaller focus group sizes for health research, especially when sensitive topics are discussed...considering 4 to 6 persons to be optimal.” The aggregation of the individual interviews, originally a separate research project, further complemented the corpus of qualitative data and filled gaps by including the primary care physicians involved in phase B of the project.

The fourth limitation is that we did not include local and regional managers of primary care practices, an essential stakeholder category, as participants in this trial. As they deeply understand the work process in the practices, we may have missed crucial insights from this group. The fifth limitation concerns the study’s transferability. Although the researchers assessed the sample and the corpus for analysis as satisfactory, the settings are specific to 1 practice in phase A and 1 region of Rio de Janeiro’s primary care practices in phase B when considering the qualitative data collection. This may limit how the results can be generalized to other parts of the city or further geographic spaces and contexts. Regarding the quantitative methods, the large proportion of missing follow-up data undermines the outcome assessment. Therefore, all conclusions about the quantitative analysis must be seen as a trend, not a significant result. The findings are exploratory and should be interpreted cautiously. According to the CONSORT recommendations for feasibility trials and pilot studies [51], determining and attaining an adequate sample size is out of the scope of feasibility studies as the objective is not to draw statistical significance of power; otherwise, the subsequent trial would not be necessary. In any case, we relied on this result to anticipate and develop mitigation strategies for the ongoing trial, such as the active recruitment of patients based on hospital discharge lists and the inclusion of a more robust research team to ensure a higher participant recruitment success rate and better data collection [46].

### Harms and Risks

The intervention in this study inflicted minimal risk or unintended effects on the participants. However, we considered the patients’ concerns about being blocked from accessing specialized consultations.

### Conclusions

Considering the described adaptations, this study showed that it is feasible to offer telehealth support from cardiologists to primary care physicians to treat patients with heart failure in the community setting in Rio de Janeiro, Brazil. Primary care physicians found it valuable and feasible but pointed to hardships in engagement due to work overload. Patients were receptive, although they might feel unsafe if they do not have direct access to a cardiologist. Cardiologists evaluated the intervention as an attainable opportunity to connect primary and specialized care. Considering the needed modifications in recruitment and educational strategies, the intervention was assessed as suitable for the clinical trial.

## Abbreviations

BRAHIT: Brazilian Heart Insufficiency with Telemedicine
CONSORT: Consolidated Standards of Reporting Trials
GDMT: guideline-directed medical therapy
HFrEF: heart failure with reduced ejection fraction
INC: National Institute of Cardiology
RAAS-I: renin-angiotensin-aldosterone system inhibitors
REDCap: Research Electronic Data Capture
SGLT-2: sodium-glucose cotransporter 2

## Data Availability

The datasets generated or analyzed during this study are not publicly available due to personal data protection policies but are available from the corresponding author on reasonable request.
